# Supplementary material for: Exploring reported population differences in Norway lobster (Nephrops norvegicus) in the Pomo Pits region of the Adriatic Sea using genome-wide markers
Source: PeerJ. 2024 Oct 21;12:e17852. doi: 10.7717/peerj.17852 (PMC11500701; doi:10.7717/peerj.17852)
Supplement: Supplemental Information 1 [file peerj-12-17852-s001.pdf]

Supplementary Material: S1

Genome-wide markers reveal panmixia of Norway lobster (*Nephrops norvegicus*) stocks in the Adriatic Sea

Tom L. Jenkins, Michela Martinelli, Jamie R. Stevens

2023-10-18

Model carapace length

This document contains the results of modelling carapace length (mm) as a function of site and sex of Norway lobsters.

```r
# Load packages
library(ggplot2)
library(readr)
library(dplyr, warn.conflicts = FALSE)
library(performance)
library(see)
```

Read in and prepare data

```r
# Read in carapace length data
carapace_df <- read_csv("./Data/growth_data_all_samples_collected.csv",
                        show_col_types = FALSE)
carapace_df
```

```
## # A tibble: 339 x 6
##    Ind_ID Site  Sea      Pomo  Sex   Carapace_length_mm
##    <chr>  <chr> <chr>    <chr> <chr>              <dbl>
##  1 SP90   Pom1  Adriatic Yes   M                     43
##  2 SP91   Pom1  Adriatic Yes   F                     30
##  3 SP92   Pom1  Adriatic Yes   M                     35
##  4 SP93   Pom1  Adriatic Yes   M                     39
##  5 SP94   Pom1  Adriatic Yes   M                     28
##  6 SP95   Pom1  Adriatic Yes   M                     28
##  7 SP96   Pom1  Adriatic Yes   F                     28
##  8 SP97   Pom1  Adriatic Yes   M                     24
##  9 SP98   Pom1  Adriatic Yes   M                     29
## 10 SP99   Pom1  Adriatic Yes   M                     30
## # i 329 more rows
```

```r
# Convert sites column to factor and reorder sites
site_order <- c("Cly","17I","18II","Anc","Cgg","Pom1","Pom2","Pom3")
carapace_df$Site <- factor(carapace_df$Site, levels = site_order)
```

```r
# Convert pomo and sex column to factor
carapace_df$Pomo <- factor(carapace_df$Pomo)
carapace_df$Sex <- factor(carapace_df$Sex, labels = c("Female","Male"))
```

```r
# Boxplot grouped by sex (male or female)
fig1C <- ggplot(data=carapace_df, aes(x=Site, y=Carapace_length_mm))+
  geom_boxplot(aes(fill=Sex), position = position_dodge(0.7))+
  # geom_violin(aes(fill=Sex), position = position_dodge(0.7))+
  scale_fill_manual(values= c("#dd1c77","royalblue"),
                    labels= c("Female","Male"))+
  ylab("Carapace length (mm)\n")+
  ggtitle("Carapace length variation")+
  theme_bw()+
  theme(plot.title = element_text(hjust = 0.5, size = 15),
        axis.title.x = element_blank(),
        axis.title.y = element_text(size = 12),
        axis.text = element_text(size = 12, colour = "black")
  )
fig1C
```

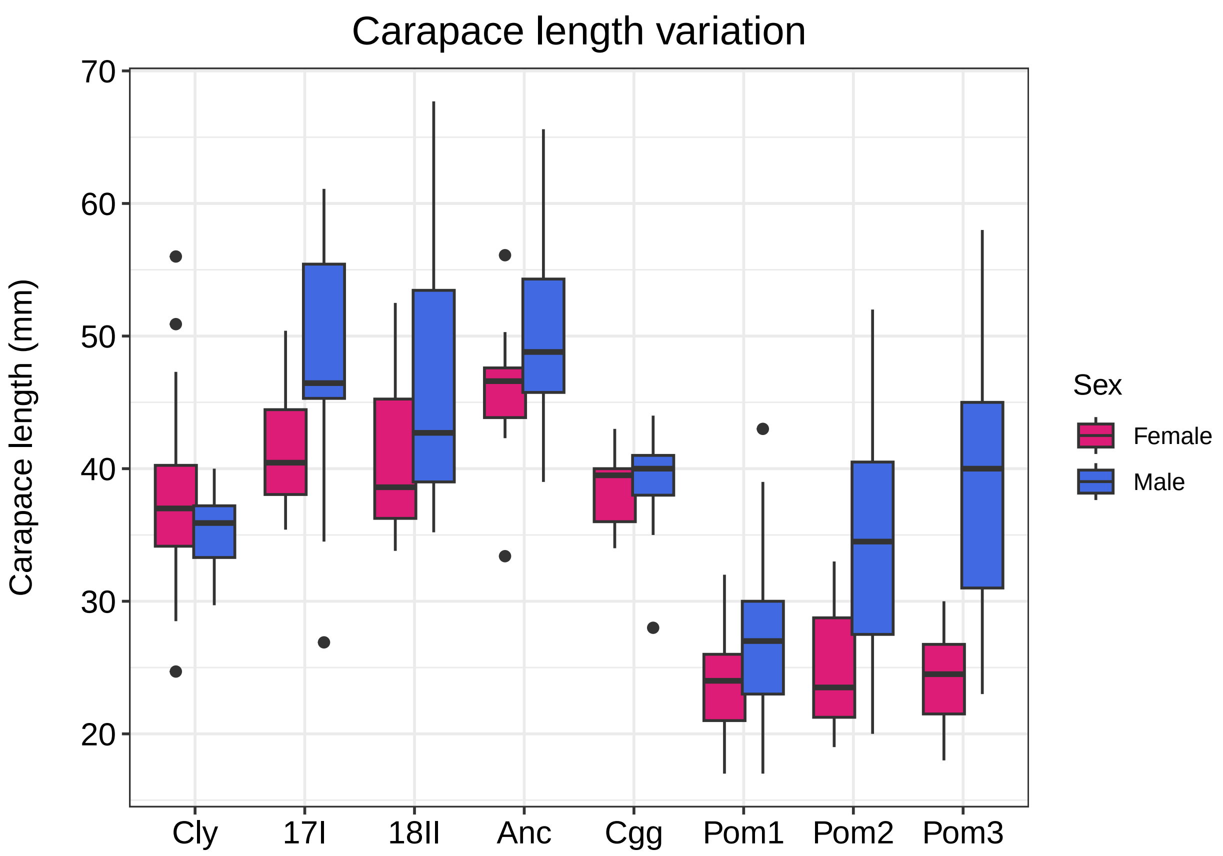

```r
# Filter data.frame to only include Adriatic sites
carapace_df <- filter(carapace_df, Sea == "Adriatic")
```

```r
# Male average carapace length: Pomo Pit
male_pomo <- carapace_df |>
  filter(.data = _, Pomo == "Yes" & Sex == "Male") |>
  pull(.data =_, Carapace_length_mm) |>
  median(x = _) |>
  round(x = _, digits = 1)

# Male average carapace length: Outside Pomo Pit
male_outside <- carapace_df |>
  filter(.data = _, Pomo == "No" & Sex == "Male") |>
  pull(.data =_, Carapace_length_mm) |>
  median(x = _) |>
  round(x = _, digits = 1)

# Female average carapace length: Pomo Pit
female_pomo <- carapace_df |>
  filter(.data = _, Pomo == "Yes" & Sex == "Female") |>
  pull(.data =_, Carapace_length_mm) |>
  median(x = _) |>
  round(x = _, digits = 1)

# Female average carapace length: Outside Pomo Pit
female_outside <- carapace_df |>
  filter(.data = _, Pomo == "No" & Sex == "Female") |>
  pull(.data =_, Carapace_length_mm) |>
  median(x = _) |>
  round(x = _, digits = 1)

# Print median averages
tibble(
  `group` = c("Male Pomo Pit", "Female Pomo Pit", "Male Outside", "Female Outside"),
  `median carapace length (mm)` = c(male_pomo, female_pomo, male_outside, female_outside),
  `n` = c(95, 77, 73, 62)
)
```

```
## # A tibble: 4 x 3
##   group           `median carapace length (mm)`     n
##   <chr>                                    <dbl> <dbl>
## 1 Male Pomo Pit                               28    95
## 2 Female Pomo Pit                             24    77
## 3 Male Outside                                46    73
## 4 Female Outside                              40    62
```

Modelling

```r
# ANOVA: model carapace length as a function of site (factor)
model1 <- lm(Carapace_length_mm ~ Pomo, data = carapace_df)
```

```r
# Check model assumptions
check_model(model1)
```

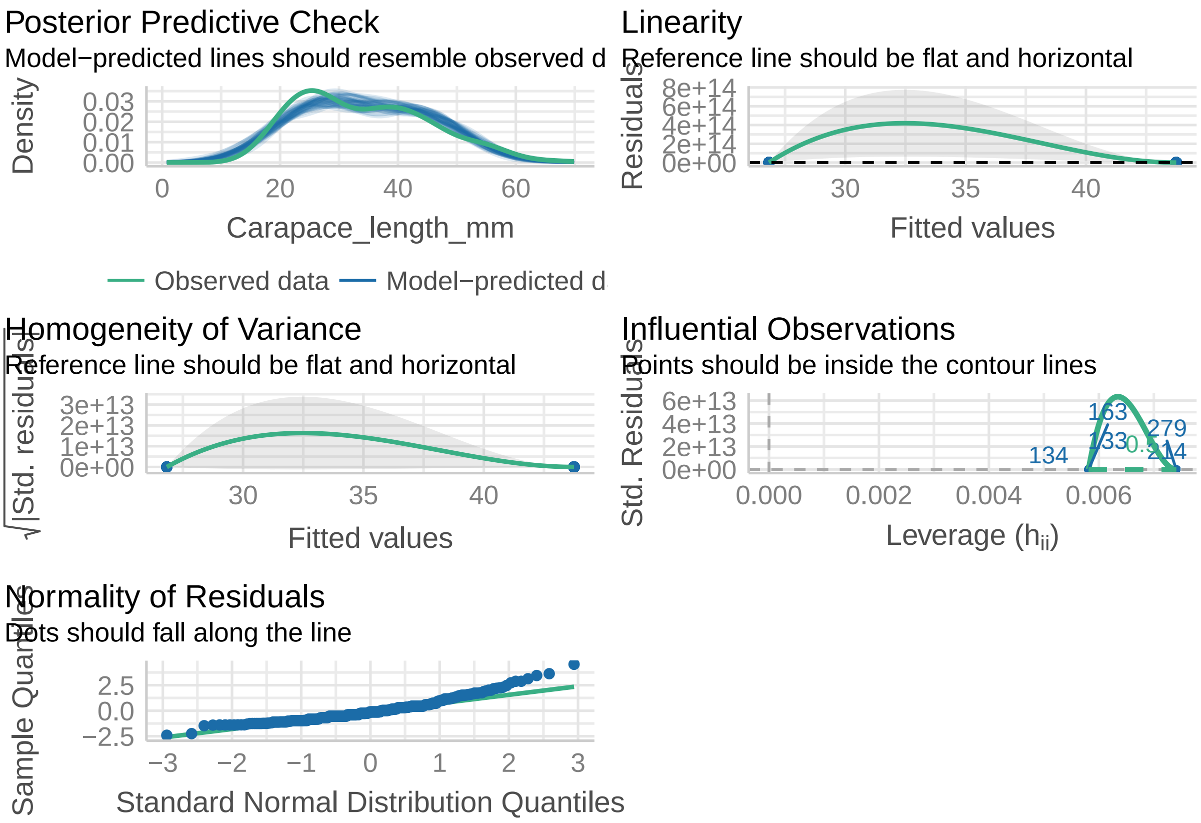


```r
# ANOVA: model carapace length as a function of site (factor) and sex (factor)
model2 <- lm(Carapace_length_mm ~ Pomo + Sex, data = carapace_df)
```

```r
# Check model assumptions
check_model(model2)
```

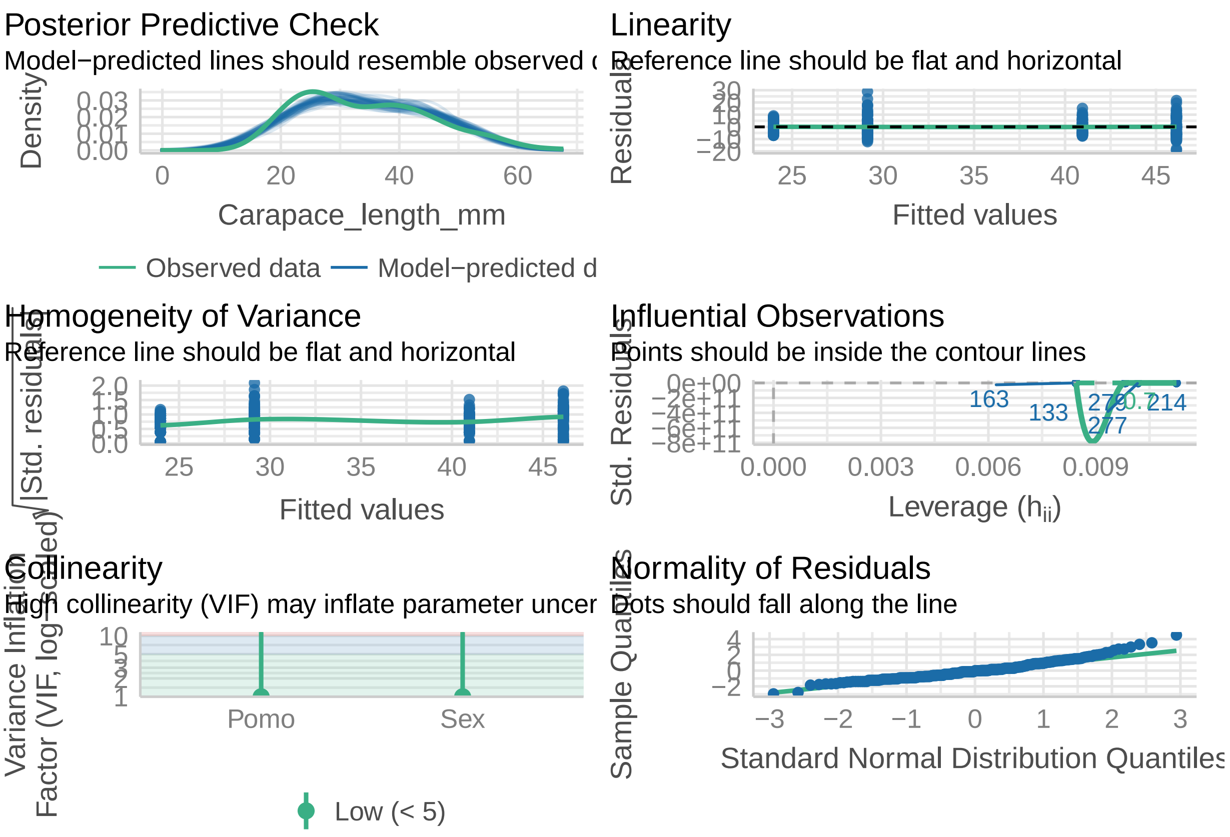


```r
# Compare models
compare_performance(model1, model2, rank = TRUE, metrics = "common")
```

```
## # Comparison of Model Performance Indices
##
## Name   | Model |    R2 | R2 (adj.) |  RMSE | AIC weights | BIC weights | Performance-Score
## -------------------------------------------------------------------------------------------
## model2 |    lm | 0.640 |     0.637 | 6.590 |       1.000 |       1.000 |           100.00%
## model1 |    lm | 0.585 |     0.584 | 7.074 |    9.63e-10 |    6.20e-09 |             0.00%
```

```r
# Best model summary
summary(model2)
```

```
##
## Call:
## lm(formula = Carapace_length_mm ~ Pomo + Sex, data = carapace_df)
##
## Residuals:
##      Min       1Q   Median       3Q      Max
## -19.2227  -4.9555  -0.1446   3.0836  28.8554
##
## Coefficients:
##             Estimate Std. Error t value Pr(>|t|)
## (Intercept)  40.9555     0.7025  58.301  < 2e-16 ***
## PomoYes     -16.9781     0.7615 -22.295  < 2e-16 ***
```

```
## SexMale       5.1672     0.7594   6.805  5.4e-11 ***
## ---
## Signif. codes:  0 '***' 0.001 '**' 0.01 '*' 0.05 '.' 0.1 ' ' 1
##
## Residual standard error: 6.623 on 304 degrees of freedom
## Multiple R-squared:  0.6398, Adjusted R-squared:  0.6374
## F-statistic:   270 on 2 and 304 DF,  p-value: < 2.2e-16
```
